# MRI in female pelvis: an ESUR/ESR survey

**DOI:** 10.1186/s13244-021-01152-w

**Published:** 2022-03-28

**Authors:** Stephanie Nougaret, Yulia Lakhman, Sophie Gourgou, Rahel Kubik-Huch, Lorenzo Derchi, Evis Sala, Rosemarie Forstner, the European Society of Radiology (ESR) and the European Society of Urogenital Radiology (ESUR)

**Affiliations:** 1 IRCM, Montpellier Cancer Research Institute, 208 Ave des Apothicaires, 34295 Montpellier, France; 2 Department of Radiology, Montpellier Cancer Institute, INSERM, U1194, University of Montpellier, 208 Ave des Apothicaires, 34295 Montpellier, France; 3 Body Imaging Service, Department of Radiology, Memorial Sloan Kettering Cancer Center, New York, NY USA; 4 Department of Statistics, Montpellier Cancer Institute, University of Montpellier, 208 Ave des Apothicaires, 34295 Montpellier, France; 5 Department of Radiology, Kantonsspital Baden, Baden, Switzerland; 6 Universita Degli Studi Di Genova, Via A Pastore 1, 16132 Genoa, Italy; 7 Department of Radiology, Cambridge Biomedical Campus, Box 218, Cambridge, CB2 0QQ UK; 8 Department of Radiology, Universitätsklinikum Salzburg, PMU, Müllner Hauptstr. 48, 5020 Salzburg, Austria

**Keywords:** Gynaecology, Magnetic resonance imaging, Practice guideline, Radiologists, Survey and questionnaires

## Abstract

**Objectives:**

While magnetic resonance imaging (MRI) is considered the gold standard for the imaging of female pelvis, there is an ongoing debate about the most appropriate indications and optimal imaging protocols. The European Society of Urogenital Radiology (ESUR) launched a survey to evaluate the current utilization of female pelvic MRI in clinical practice.

**Methods:**

The ESUR female imaging subgroup developed an online survey that was then approved by the ESR board and circulated among the ESR members. The questions in the survey encompassed training and experience, indications for imaging and MR imaging protocols, reporting styles and preferences. The results of the survey were tabulated, and subgroups were compared using *χ*^2^ test.

**Results:**

A total of 5900 ESR members with an interest in both MRI and female pelvic imaging were invited to participate; 840 (14.23%) members completed the survey. Approximately 50% of respondents were academic radiologists (50.6%) and nearly 60% women (59.69%). One third of the respondents were subspecialized in Gynecological imaging. Nearly half of the survey participants were aware of the presence of ESUR guidelines for imaging of the female pelvis (47.1%). The adoption of the ESUR recommendations was higher among subspecialized and/or academic and/or senior and/or European radiologists compared to all others. The current ESUR recommendations about female pelvic MRI protocols were generally followed. However wide variations in practice were identified with respect to the use of contrast media.

**Conclusion:**

Female pelvic MRI protocol was generally following the ESUR recommendations, especially among subspecialized and academic radiologists. However, the fact that they are followed by only half of the participants highlights the need for wider awareness of these recommendations*.*

## Key points

- The current ESUR recommendations about female pelvic MRI protocols were generally followed.
- ESUR guidelines are used by 48% of the radiologists participating in this survey which highlights the need for greater awareness of these recommendations.
- Subspeciality and/or academic and/or senior and/or European radiologists are most familiar with and are most likely to use these guidelines.

## Introduction

Magnetic resonance imaging (MRI) has become the main modality to establish the diagnosis and guide management of patients with gynecological diseases. In oncology, for example, MRI has been incorporated into various clinical guidelines to assess the tumor extent (NCCN, ESMO, FIGO, ESUR, ACR…) [1, 2]. However, the indications for MRI of female pelvis vary across societies (NCCN, ESMO, FIGO, ESUR, ACR…) due to regional clinical preferences. Another factors contributing to inter-institutional and international variations are relatively high cost of MRI, limited availability in some locations, and potential reimbursement-related challenges. Further, wide variations exist with respect to MR image acquisition and interpretation. The European Society of Urogenital Radiology (ESUR) published several imaging guidelines including recent updates in the last four years in order to make practice more uniform and up to date among centers and radiologists [3–9]. These updates were prompted by the recent advances in MRI, including increased implementation of functional imaging, i.e. diffusion weighted Imaging (DWI) and dynamic contrast-enhanced MRI (DCE-MRI) [10]. For example, the recent ESUR guideline for the assessment of sonographically indeterminate adnexal masses recommended the use of contrast-enhanced T1WI, preferably using DCE-MRI and time intensity curves for the improved characterization [7]. The use of DWI is now recommended for the evaluation of all gynaecological malignancies [5, 6, 11, 12]. However, while there are consensus guidelines, their knowledge and implementation in clinical practice among radiologists is unknown.

Therefore, the ESUR female pelvis imaging group decided to conduct a survey among the members of the European Society of Radiology (ESR) to gather representative data on current female pelvic MRI practice, patterns of pelvic MRI requests, MRI protocols and to determine how widely these ESUR guidelines have been implemented in routine clinical practice among ESR members.

## Materials and methods

### Survey design and distribution

Two board certified radiologists (S.N. and R.F.) with 8 and over 25 years of experience in female pelvic imaging developed the survey. It was comprised of 33 questions which included general demographic information, professional training and experience, annual volume of female pelvic MRI examinations; indications and technical details of MRI examinations, and, lastly, reporting habits and preferences. Some questions asked to select all applicable answers; there was no requirement to answer all questions prior to submission. The full questionnaire is available online under the supplement. The survey was first approved by the ESUR female pelvic imaging working group and then by both the ESUR board and the European Society of Radiology (ESR) executive board.

The survey was published online (Survey Monkey www.surveymonkey.de) and announced by the ESUR administrative office via electronic mail. All 5,900 ESR members who previously indicated an interest in both “Gynaecology and Obstetrics” and “MRI” were invited. The survey opened online on May 7, 2019, and remained active for a 5-week period, with two email reminders sent by the ESUR office during the survey period.

### Data analysis

After the survey closed, all responses were extracted and summarized by the ESUR administrative office. In addition, subgroup analyses were performed with the focus on the degree of expertise (gynecological imaging expertise), institution type (academic center vs. other), geographic location (Europe and rest of the world), and years in practice (resident vs. senior). Qualitative variables were described by the number of observations (*n*) and their frequencies (%). The missing categories were counted. The percentages were calculated in relation with excluded missing data. The *χ*^2^ test, or Fisher's exact test were used for comparisons.

The threshold for significance was set at 5% (i.e. *p* = 0.05). Statistical analyses were performed using the R studio v4.0.0 software (2020-04-24).

## Results

Among the 5900 ESR members invited to take the survey, 840 returned the survey, i.e., a response rate of 14.23% Full results of the survey are available as supplementary data.

Internationally, countries with the higher response rates were India (*n* = 85), followed by Saudia Arabia (*n* = 32), and Pakistan (*n* = 31). Among European countries the highest number of answers (*n* = 34) were collected from Great Britain and Spain, followed by Romania (*n* = 30) and Portugal (*n* = 20).

**Fig. 1:**
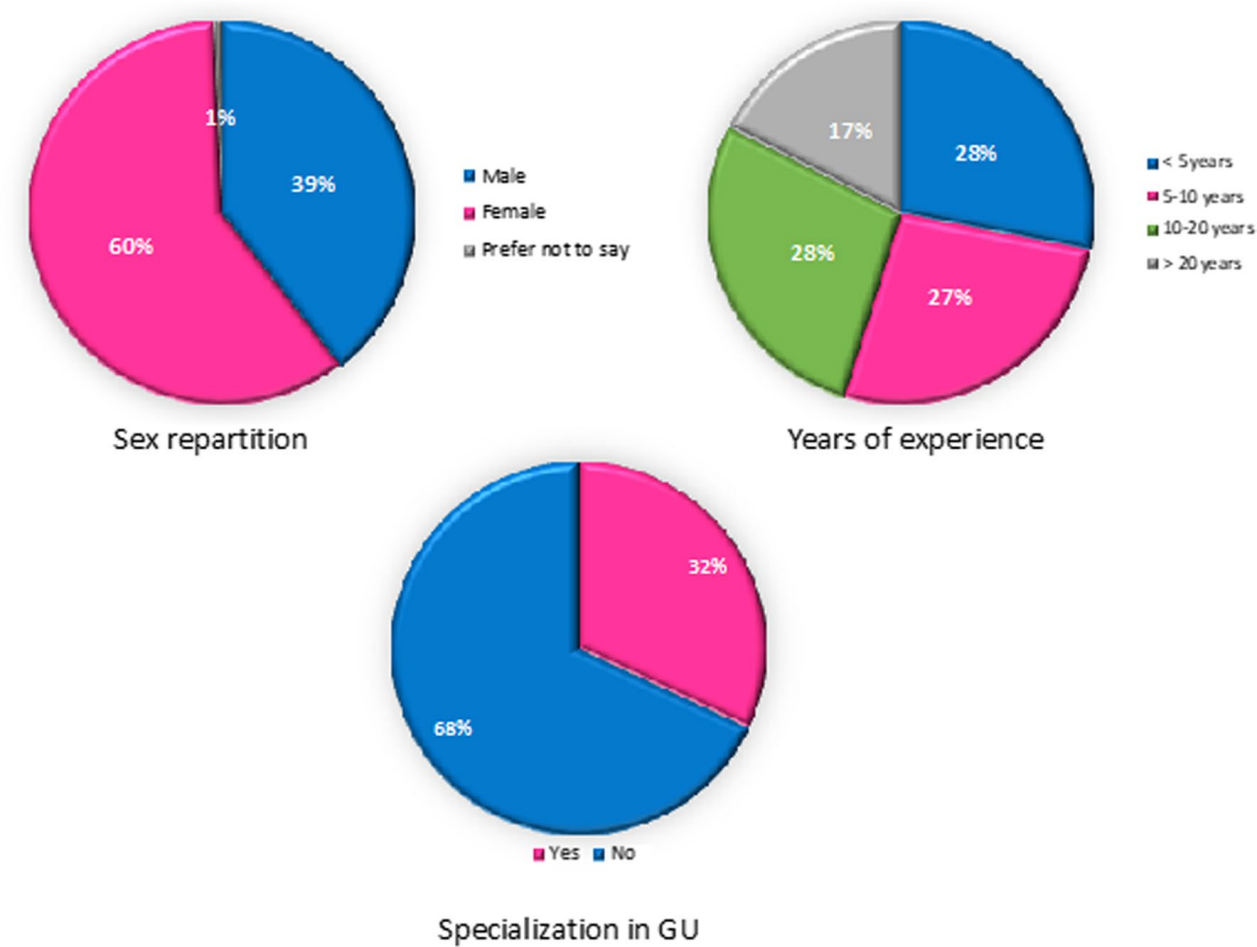
Overview of the participants

Over half of the participants were practicing in an academic setting (50.6%), nearly 60% were women (59.69%) (Fig. 1), and a third subspecialized in gynecological imaging (Fig. 1).

### Indications for female pelvic MRI

**Fig. 2:**
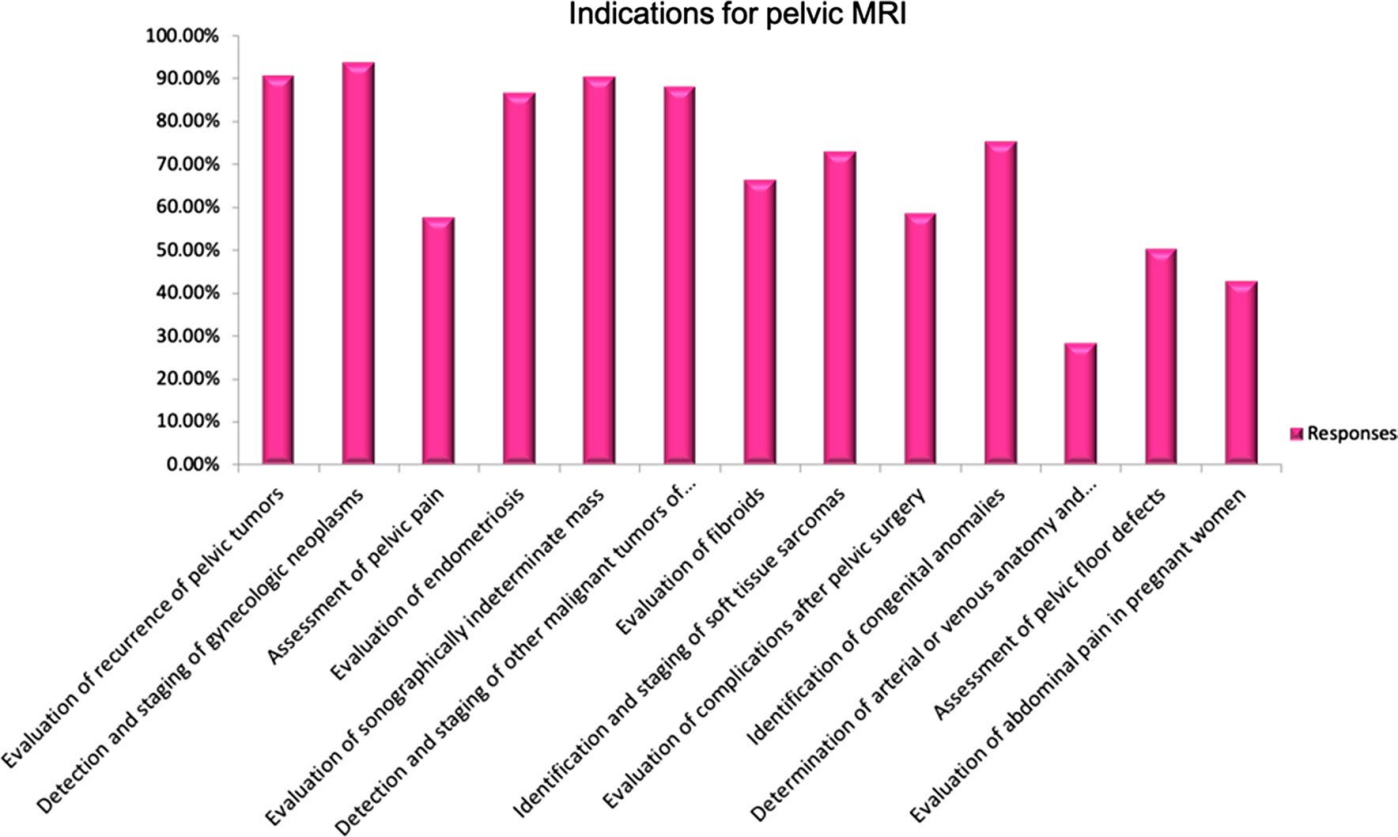
Indications for pelvic MRI

The most common indications for MRI were detection and staging of gynecologic neoplasms (80.95%) followed by evaluation of suspected or confirmed recurrent pelvic tumor (78.45%) and sonographically indeterminate adnexal mass (78.21%) (Fig. 2).

### MRI protocol

Answers regarding MRI protocols are summarized in Table 1. Most MRI examinations were performed on a 1.5 and/or 3 T units (94.04%). Most radiologists used a tailored protocol as recommended by the ESUR guidelines: T2/T1 sequence covering the paraaortic regions were performed by 70.20%. Oblique sequence perpendicular to the short axis of the uterine corpus or cervix for endometrial and cervical cancer staging, respectively, were performed by 85.23%; slice thickness ≤ 4 mm for axial or axial oblique sequence was used by 64.30%. In contrast, the use of gadolinium-enhanced T1WI FS sequence deviated from the guidelines. (Table 1). For example, only 63.5% of radiologists used contrast-enhanced MR imaging for assessment of a sonographically indeterminate adnexal masses and only 40.12% for evaluation of myometrial masses. DCE MRI was used even less. For example, only 28.93% of radiologists used DCE-MRI in the assessment of a sonographically indeterminate adnexal mass. In contrast, diffusion weighted imaging (DWI) was obtained in 41.31% of female pelvic MRI exams indicating wider adoption compared to DCE-MRI (Table 1). When DWI images were acquired, axial plane was used most often and a high *b* value of ≥ 800 was obtained by 64% of radiologists.

**Table 1:** Main result summary

	*n* = 840
Institution type	
Non-university hospital University hospital NA	393 (49.5) 425 (50.5) 22
Gender	
Male Female Prefer not to say NA	328 (39.71) 493 (59.69) 5 (0.61) 14
Experience	
< 5 years 5–10 years 10–20 years > 20 years NA	199 (45.33) 108 (24.60) 96 (21.87) 36 (8.20) 401
Indications for pelvic MRI	
Evaluation of recurrence of pelvic tumors Detection and staging of gynecologic neoplasms Assessment of pelvic pain Evaluation of endometriosis Evaluation of sonographically indeterminate mass Detection and staging of other malignant tumors of the pelvis	659 (78.45) 680 (80.95) 416 (49.52) 628 (74.76) 657 (78.21) 638 (75.95)
Eval. of fibroids Identification and staging of soft tissue sarcomas Evaluation of complications after pelvic surgery Identification of congenital anomalies Determination of arterial or venous anatomy and patency Assessment of pelvic defects Evaluation of abdominal pain in pregnant women	481 (57.26) 529 (63.00) 422 (50.23) 546 (65.00) 205 (24.40) 366 (43.57) 309 (36.78)
Use of T2/T1 sequence covering the whole paraaortic regions	
Yes No NA	483 (70.20) 205 (29.80) 152
Oblique sequence perpendicular to the axis of the uterus or cervix for endometrial or cervical cancer staging	
Yes No NA	577 (85.23) 100 (14.77) 163
Slice thickness of axial oblique sequence (< 4 mm)	
Yes No NA	395 (64.30) 221 (35.70) 224
Gadolinium T1WI FS sequence	
Yes, in every case No, never Evaluation of recurrence of pelvic tumors In case of detection of gynecologic neoplasms In case of staging of gynecologic neoplasms In case of assessment of a pelvic mass In case of evaluation of fibroids In case of identification of congenital anomalies In case of evaluation of Endometriosis In case of assessment of pelvic floor defects Other	155 (18.45) 230 (27.38) 541 (64.4) 520 (61.9) 530 (63.1) 533 (63.5) 337 (40.12) 176 (21.0) 277 (33.0) 161 (19.17) 47 (5.6)
Gadolinium cat	
No, never At least one Yes, in every case	230 (27.38) 455 (54.17) 155 (18.45)
Dynamic Contract enhanced/perfused MRI	
Yes, in every case No, never Evaluation of recurrence of pelvic tumors In case of detection of gynecologic neoplasms In case of staging of gynecologic neoplasms In case of assessment of a pelvic mass In case of evaluation of fibroids In case of identification of congenital anomalies In case of evaluation of Endometriosis In case of assessment of pelvic floor defects Other	65 (7.74) 473 (56.31) 238 (28.33) 258 (30.71) 267 (31.79) 243 (28.93) 127 (15.12) 69 (8.21) 90 (10.71) 71 (8.45) 38 (4.5)
Dynamic Contract enhanced cat	
No, never At least one Yes, in every case	473 (56.31) 302 (35.95) 65 (7.74)
DWI sequence	
Yes, in every case No, never Evaluation of recurrence of pelvic tumors In case of detection of gynecologic neoplasms In case of staging of gynecologic neoplasms In case of assessment of a pelvic mass In case of evaluation of fibroids In case of identification of congenital anomalies In case of evaluation of Endometriosis In case of assessment of pelvic floor defects Other	347(41.31) 285 (33.92) 519 (61.79) 525 (62.50) 515 (61.31) 508 (60.48) 419 (49.88) 350 (41.67) 396 (47.14) 348 (41.43) 27 (3.2)
DWI sequence cat	
No, never At least one Yes, in every case	285 (33.93) 208 (24.76) 347 (41.31)
Do you use standardized reporting?	
Yes No NA	281 (46.29) 326 (53.71) 233
Are you aware of ESUR guidelines?	
Yes No NA	290 (47.9) 315 (52.1) 235
Do you use ESUR guidelines?	
Yes No NA	290 (47.93) 315 (52.07) 235

### The use of MRI reporting guidelines

This question was not answered by 233 participants. Of 607 respondents, the standardized report was used by 46.29%. Nearly half of the responders were aware of the presence of ESUR guidelines for imaging of the female pelvis (47.1%). Among them, the reporting guidelines for staging of endometrial and cervical cancer were the most used (69.85% resp. 68.75%) (Table1).

### Subgroup analysis

Radiologists subspecialized in gynecological imaging were more likely to be familiar with the ESUR guidelines for imaging of the female pelvis. The use of the recommended axial oblique T2 sequence through the uterus or cervix in case of endometrial or cervical cancer, respectively, and a slice thickness ≤ 4 mm was significantly more used among subspecialized radiologists versus non specialists (oblique axial sequence: 92.24% by specialists vs. 81.57% by non-specialists *p* < 0.001; slice thickness ≤ 4 mm: 87.5% vs. 72.2%, *p* < 0.001). The use of ESUR guidelines was significantly more frequent among specialists versus in non-specialists (63.51% vs. 38.90%; *p* < 0.001) (Table 2). The use of DWI, contrast-enhanced imaging and DCE-MRI was significantly more frequent among sub-specialized versus general radiologists (*p* < 0.001) (Table 2).

**Table 2:** Subgroup analysis regarding expertise in gynecological imaging

	Non specialist	Specialist	*p* value
	*n* = 554	*n* = 264	
Institution type			
Non-university hospital University hospital NA	294 (53.07) 260 (46.93) 0	99 (37.50) 165 (62.50) 0	< 0.001*
Gender			
Female Male Prefer not to say NA	320 (57.76) 230 (41.52) 4 (0.72) 0	167 (63.26) 96 (36.36) 1 (0.38) 0	0.297**
Use of T2/T1 sequence covering the whole paraaortic regions			
Yes No NA	319 (70.26) 135 (29.74) 100	164 (70.09) 70 (29.91) 30	0.961*
Oblique sequence perpendicular to the axis of the uterus or cervix for endometrial or cervical cancer staging			
Yes No NA	363 (81.57) 82 (18.43) 109	214 (92.24) 18 (7.76) 32	< 0.001*
Slice thickness of axial oblique sequence			
> 4 mm ≤ 4 mm NA	110 (27.8) 286 (72.2) 158	33 (12.5) 189 (87.5) 42	< 0.001*
Do you perform Gadolinium T1WI FS sequence?			
No, never At least one Yes, in every case	161 (29.06) 289 (52.17) 104 (18.77)	46 (17.42) 167 (63.26) 51 (19.32)	0.002*
Do you perform Dynamic Contrast enhanced/perfusion MRI?			
No, never At least one Yes, in every case	347 (62.63) 170 (30.70) 37 (6.7)	131 (39.4) 132 (50.00) 28 (10.6)	< 0.001*
Do you perform DWI sequence?			
No, never At least one Yes, in every case	218 (39.4) 123 (22.2) 213 (38.4)	45 (17.0) 85 (32.2) 134 (50.8)	< 0.001*
Do you use standardized reporting?			
Yes No NA	185 (48.05) 200 (51.95) 169	96 (43.24) 126 (56.76) 42	0.252*
Do you use ESUR guidelines?			
Yes No NA	149 (38.90) 234 (61.10) 171	141 (63.51) 81 (36.49) 42	< 0.001*

*χ^2^ test; **Fisher

Women radiologist and gynecological subspecialists were more likely to practice at academic institutions (female vs. male 64.35%/35.19% in academic vs. 54.57%/44.67% in non-academic settings *p* = 0.01; specialist in gynecological vs. non specialist 38.82/61.18% in academic versus 25.19/74.81% in non-academic practice *p* < 0.001). No difference was found in term of the use of MRI protocols between academic and non-academic radiologists except for the use of DWI. The use of DWI was more frequent at academic centers compared to non-academic practices (*p* = 0.035). The use of ESUR guidelines was significantly more frequently reported by academic (54.21%) compared to non-academic radiologists (40.85%; *p* = 0.001) (Table 3). In contrast, the use of a reporting template was more frequent at non-academic practices (51.75%) compared to academic centers (41.43; *p* = 0.011) (Table 3).

**Table 3:** Subgroup analysis regarding type of institution

	Academic practice	Non academic practice	*p* value
	*n* = 434	*n* = 395	
Gender			
Female Male Prefer not to say NA	278 (64.35) 152 (35.19) 2 (0.46) 2	215 (54.57) 176 (44.67) 3 (0.76) 1	0.010**
Specialist in gynecological imaging			
Specialist Non specialist NA	165 (38.82) 260 (61.18) 9	99 (25.19) 294 (74.81) 2	< 0.001*
Use of T2/T1 sequence covering the whole paraaortic regions			
Yes No NA	248 (69.66) 108 (30.34) 78	235 (70.78) 97 (29.22) 63	0.748*
Oblique sequence perpendicular to the axis of the uterus or cervix for endometrial or cervical cancer staging			
Yes No NA	309 (87.78) 43 (12.22) 82	268 (82.46) 57 (17.54) 70	0.051*
What is the slice thickness of your axial or axial oblique sequence?			
> 4 mm ≤ 4 mm NA	115 (35.49) 209 (64.51) 110	106 (35.93) 189 (64.07) 100	0.909*
Do you perform Gadolinium T1WI FS sequence?			
No, never At least one Yes, in every case	115 (26.5) 235 (54.1) 84 (19.4)	104 (26.3) 220 (55.7) 71 (18.0)	0.859*
Do you perform dynamic contrast enhanced/perfusion MRI?			
No, never At least one (%) Yes, in every case	231 (53.2) 170 (39.2) 33 (7.6)	231 (58.5) 132 (33.4) 32 (8.1)	0.227*
Do you perform DWI sequence?			
No, never At least one Yes, in every case	129 (29.7) 106 (24.4) 199 (45.9)	145 (36.7) 102 (25.8) 148 (37.5)	0.035*
Do you use standardized reporting?			
Yes No NA	133 (41.43) 188 (58.57) 113	148 (51.75) 138 (48.25) 109	0.011*
Do you use ESUR guidelines?			
Yes No NA	174 (54.21) 147 (45.79) 113	116 (40.85) 168 (59.15) 111	0.001*

*χ^2^ test; **Fisher

We were interested to determine if there was a difference between Europe, where the guidelines originate from ESR members of non-European countries. Thus, for comparing the practice among radiologists worldwide, due to the relative low number of respondents (or relatively low number of respondents outside of Europe) worldwide the comparisons were made between Europe (*n* = 376) and other countries (*n* = 464). Interestingly, large differences in terms of imaging protocol were seen. The use of an oblique plane perpendicular to the long axis of the uterus or cervix, a slice thickness < 4 mm, the use of DWI, and DCE-MRI were significantly more frequent in Europe compared the other countries (Table 4). The use of ESUR guidelines was significantly higher among European (63.18%) compared to non-European radiologists (35.06%) (*p* < 0.001). In contrast, the use of a reporting template was more frequent in non-European (58.05%) compared to European centers (32.37%; *p* < 0.001) (Table 4).

**Table 4:** Subgroup analysis regarding localization

	Other	Europe	*p* value
	*n* = 464	*n* = 376	
Establishment type			
Academic Non Academic NA	209 (45.93) 246 (54.07) 9	225 (60.16) 149 (39.84) 2	< 0.001*
Gender			
Female Male Prefer not to say NA	259 (57.17) 192 (42.38) 2 (0.44) 11	234 (62.73) 136 (36.46) 3 (0.80) 3	0.183**
Specialty			
Specialist Non-specialist NA	117 (26.06) 332 (73.94) 15	147 (39.84) 222 (60.16) 7	< 0.001*
Do you also use a T2/T1 sequence that covers the whole paraaortic regions?			
Yes No NA	291 (75.19) 96 (24.81) 77	192 (63.79) 109 (36.21) 75	0.001*
Do you use axial oblique sequence perpendicular to the axis of the uterus or cervix			
Yes No NA	302 (79.47) 78 (20.53) 84	275 (92.59) 22 (7.41) 79	< 0.001*
What is the slice thickness of your axial or axial oblique sequence?			
> 4 mm ≤ 4 mm NA	139 (41.00) 200 (59.00) 125	82 (29.29) 198 (70.71) 96	0.002*
Do you use standardized reporting?			
Yes No NA	191 (58.05) 138 (41.95) 135	90 (32.37) 188 (67.63) 99	< 0.001*
Do you use ESUR guidelines?			
Yes No NA	115 (35.06) 213 (64.94) 136	175 (63.18) 102 (36.82) 99	< 0.001*
Do you perform Gadolinium T1WI FS sequence?			
No, never At least one Yes, in every case	129 (27.80) 249 (53.70) 86 (18.50)	101 (26.90) 206 (54.80) 69 (18.4)	0.942
Do you perform Dynamic Contrast enhanced/perfusion MRI?			
No, never At least one Yes, in every case	282 (60.8) 149 (32.1) 33 (7.1)	191 (50.8) 153 (40.7) 32 (8.5)	0.015
Do you perform DWI sequence?			
No, never At least one Yes, in every case	182 (39.2) 118 (25.4) 164 (35.3)	103 (27.4) 90 (23.9) 183 (48.7)	< 0.001*

*χ^2^ test; **Fisher

Regarding radiologists’ experience, the use of ESUR guidelines were significantly more likely among senior radiologists (50.66%) compared to less experienced radiologists (39.60%) (*p* = 0.019). The use of an oblique plane perpendicular to the long axis of the uterus or cervix, DWI, contrast-enhanced imaging, and DCE-MRI were significantly more frequent among senior compared to junior radiologists (Table 5).

**Table 5:** Subgroup analysis regarding Radiology practice

	Resident	Senior	*p* value
	*n* = 229	*n* = 593	
Establishment type			
Academic Non Academic NA	106 (46.29) 123 (53.71) 0	288 (48.57) 305 (51.43) 0	0.558*
Gender			
Female Male Prefer not to say NA	148 (64.63) 80 (34.93) 1 (0.44) 0	342 (57.67) 247 (41.65) 4 (0.67) 0	0.158**
Specialty			
Specialist Non-specialist NA	29 (12.72) 199 (87.28) 1	235 (39.83) 355 (60.17) 3	< 0.001*
Do you also use a T2/T1 sequence that covers the whole paraaortic regions?			
Yes No NA	134 (72.83) 50 (27.17) 45	349 (69.25) 155 (30.75) 89	0.363*
Do you use axial oblique sequence perpendicular to the axis of the uterus or cervix			
Yes No NA	145 (80.11) 36 (19.89) 38	432 (87.10) 64 (12.90) 97	0.023*
What is the slice thickness of your axial or axial oblique sequence?			
> 4 mm < 4 mm NA	59 (38.56) 94 (61.44) 76	162 (34.69) 305 (65.31) 126	0.385
Do you use standardized reporting?			
Yes No NA	71 (47.33) 79 (52.67) 79	210 (45.95) 247 (54.05) 136	0.768*
Do you use ESUR guidelines?			
Yes No NA	59 (39.60) 90 (60.40) 80	231 (50.66) 225 (49.34) 37	0.019*
Do you perform Gadolinium T1WI FS sequence?			
No, never At least one Yes, in every case	78 (34.06) 110 (48.04) 41 (17.90)	134 (22.60) 345 (58.18) 114 (19.22)	0.003*
Do you perform Dynamic Contrast enhanced/perfusion MRI?			
No, never At least one Yes, in every case	141 (61.57) 69 (30.13) 19 (8.30)	314 (52.95) 233 (39.29) 46 (7.76)	0.048*
Do you perform DWI sequence?			
No, never At least one Yes, in every case	91 (39.74) 49 (21.40) 89 (38.86)	176 (29.68) 159 (26.81) 258 (43.51)	0.019*

*χ^2^ test **Fisher

## Discussion

Nearly half of the radiologists indicated that they were aware of one or more ESUR guidelines. The use of ESUR guidelines was highest among were Senior, academic, GU subspecialized, or European radiologists. The highest use of these guidelines among these subgroups can be explained by recent publications in major radiological journals, educational activities and presentations at subspeciality meetings in Europe. The lower rates among radiologists working in non-academic institutions or among junior radiologists points out the need for more education and teaching. Most of these guidelines are made available on the internet by open-access or can be retrieved from the ESUR homepage (https://www.esur.org/esur-guidelines/female-pelvis/).

In our study, the use of oblique axial imaging planes and thin slice thickness was adopted by most respondents for staging of cervical and endometrial cancer [5]. This is important, these imaging planes facilitate accurate tumor staging and optimal treatment planning [13]. In endometrial cancer depth of myometrial invasion is an important factor for risk stratification and clinical decision making about the need for lymphadenectomy [13–16]. In general, this may be challenging to assess particularly in equivocal cases or in the setting of co-existing benign lesions like leiomyomas and adenomyosis [17]. In cervical cancer presence of parametrial invasion warrants chemoradiation [18, 19]. The differentiation of subtle parametrial invasion (2b) from full stromal invasion (1b) requires correct angulation to exactly define the outer contour of the cervix and the interface with adjacent parametria [12]. Use of DWI was adopted by more than two thirds of the survey participants. Thus, this study confirms that DWI has become an integral component of female pelvic MR imaging. Furthermore, when the quality is adequate, DWI can substitute contrast enhanced imaging. e.g. in endometrial cancer or serve as an alternative when contrast media should be avoided, e.g. in pregnancy [20, 21]. DWI can also improve vizualisation of lymph nodes and peritoneal deposits [22]. In contrast, the use of contrast-enhanced imaging varied among radiologists worldwide. While contrast enhanced MRI was performed by 60% of radiologists for evaluation of recurrence, for staging and characterization of sonographycally indeterminate masses, there was a variety of the type of technique used. Contrast-enhanced MRI helps to differentiate tumor from non-neoplastic solid lesions, such as clots or debris within an adnexal mass. The updated ESUR guidelines recommend the use of contrast- enhanced MRI for characterization of indeterminate adnexal masses and also encourage the use of DCE-MRI [7]. Recently, the value of DCE-MRI was highlighted by the findings of a large prospective multicentre study with 1194 patients analysed [10].

Our survey showed that in clinical practice DCE-MRI is little used among radiologists interpreting female pelvic MRIs. In detail, it was never performed by 37% of radiologists and was used for adnexal mass characterization by less than 30%. DCE-MRI requires rapid image acquisition and post-processing software that may not be always available. The role of DCE-MRI is still debated and may be of diagnostic benefit only for selected cases, e.g. in differentiation of borderline tumours and invasive cancers or in the analysis of the contrast enhancement pattern to diagnose rare benign tumours. Future area of research will include change in patient management using DCE sequence and the role of non contrast studies. A recent retrospective study including 350 adnexal masses showed that expert radiologists in pelvic MRI were able to correctly diagnose adnexal masses without contrast media with high accuracy [23]. The selective use of gadolinium-based contrast media may become an increasing important issue due to gadolinium deposition in tissues. e.g. in the brain [24, 25]. However, for now, there is no currently adverse clinical outcome from this finding and adnexal MRI caracterisation usually requires a single exam and not multiple follow ups.

In addition, DCE plays a central role in the recently published O-RADS MRI risk stratification system for ovarian/adnexal masses [10, 26].

Finally, the high percentage of almost 50% of standardised reporting in clinical routine may have been biased due to the response rate of 72% for this question. Standardized reporting is rendered both in academic and non-academic but is more commonly performed in non-academic institution. This may also underline the effects of initiatives to globally standardize radiological imaging and reporting [27–29]. In this context emphasis must be put on developing a universally useable and accepted terminology (lexicon) for these reports. For ovarian mass characterisation such a lexicon has recently been published, but further effort is needed [30, 31]. Consistent technique and image quality (e.g. slice thickness and DWI *b* values) is not only of utmost importance to provide standardize imaging technique but also for exploiting this information with techniques of radiomics and machine learning algorithms. This also facilitates comparison of findings across different institutions [32]*.*

Our survey has some limitations. First, as expected with any survey, response rate was low (14%), even though many responses were received. Second, the survey was sent to radiologists associated with the ESR/ESUR (even though many were from outside Europe), who are likely to be familiar with European practice in Radiology and so they may represent a selected group. Owing to the topic of a subspecialized area in Radiology, it is understable but unavoidable to introduce a bias in comparison with general radiologists who also perform these MRI examinations.

Although this survey shows that radiologists worldwide perform female pelvic MRI studies with a technique and indications that are generally in line with the recommendations of the ESUR, barriers and opportunities to improve the knowledge of and adherence to guidelines warrant consideration [33]. Guidelines need to be practical and easily to adopt, they should be clear and not too long and should be easily accessable. Future update of existing guidelines or new guidelines can benefit from this information.

## Data Availability

The datasets are not publicly available but would be made available on reasonable request.

## Abbreviations

DCE: Dynamic contrast-enhanced
DCE-MRI: Dynamic contrast-enhanced MRI
DWI: Diffusion weighted imaging
ESR: European society of radiology
ESUR: European society of urogenital radiology
GU: Genitourinary
MRI: Magnetic resonance imaging
